# Supplementary material for: Neurotrophin-3 regulates ribbon synapse density in the cochlea and induces synapse regeneration after acoustic trauma
Source: eLife. 2014 Oct 20;3:e03564. doi: 10.7554/eLife.03564 (PMC4227045; doi:10.7554/eLife.03564)
Supplement: Source code 1. — Amira and Blob Projection software. DOI: http://dx.doi.org/10.7554/eLife.03564.015 [file elife03564s001.zip › 5281_1_supp_83813_ndfs9t(1)/Manual for amira and blob projection.docx]

**Amira program**

Create folder, put .lsm files and the script file (.hx)

Open Amira, drag the script file to the Amira window, it converts all the .lsm to

1. Amira mesh file
2. Xy, xz and yz

Open the mesh file, Right click Display, projection view. Remove xz, yz view at the bottom (Properties)

Right click compute, volume edit, right click compute, connected components

(Bottom, adjust intensity/size, click on region field/spreadsheet)

Region field: Isosurface to display the selected ribbons, unclick compactify.

Spreadsheet: save data as txt (provides counts)

(VolumeEdit to remove nuclei)

Individual channel, right click “save data as” amira mesh file.

**Blob projection Program**

Select red channel Amira mesh file

Select green channel Amira mesh file

Select blob either red or green (the spreadsheet).

Adjust radius, show grid and run. Save result image (extension follow the channel spreadsheet name, blot[v]), check colocalization by photoshop.

Repeat with spreadsheet of the other channel.
