# Supplementary material for: Neurotrophin-3 regulates ribbon synapse density in the cochlea and induces synapse regeneration after acoustic trauma
Source: eLife. 2014 Oct 20;3:e03564. doi: 10.7554/eLife.03564 (PMC4227045; doi:10.7554/eLife.03564)
Supplement: Source code 1. — Amira and Blob Projection software. DOI: http://dx.doi.org/10.7554/eLife.03564.015 [file elife03564s001.zip › 5281_1_supp_83813_ndfs9t(1)/WARNING.docx]

WARNING: This is not a piece of stand-alone software.  It was developed as part of a suite of scripts and procedures to analyze a specific type of confocal data acquired in a stereotyped way on a specific type of Confocal.  Specifically, **it will only function you use a Leica SP5 and Amira 5.2.**

It will only function if:

1) you acquire the z-stacks on a Leica SP5 and store exactly one z-stack per “.lif” file

2) you then run an Amira script (a separate piece of code) that unpacks the z-stack, reads the Leica calibration files embedded in the .lif file, and produces an Amira “mesh” (“.am” file) of the 3-D voxel space. A different Amira script would be required for each different brand and model of confocal microscope.

3) you then open the Amira mesh in Amira, and run the connected components function to find all the synaptic elements in a particular confocal channel by specifying the arbitrary pixel value that you determine does the best job of finding all the elements you are interested in.

This is an interactive process that requires learning other display features in Amira, so that you can see the components that the connected components function has “found”. Once satisfied that the criterion is “appropriate”, you then export a spreadsheet file in Amira that contains the x,y,z, coordinates of all the connected components.  You can only use Amira 5.2, because any other version of Amira packages this output data differently enough that the text parsing code in the Custom Software will not run.

 4) You place one Amira mesh and one connected components output text file in a folder together

 5) Then, and only then, are you in a position to run the Custom Software.
