# Supplementary figures and images for: Neurotrophin-3 regulates ribbon synapse density in the cochlea and induces synapse regeneration after acoustic trauma
Source: eLife. 2014 Oct 20;3:e03564. doi: 10.7554/eLife.03564 (PMC4227045; doi:10.7554/eLife.03564)

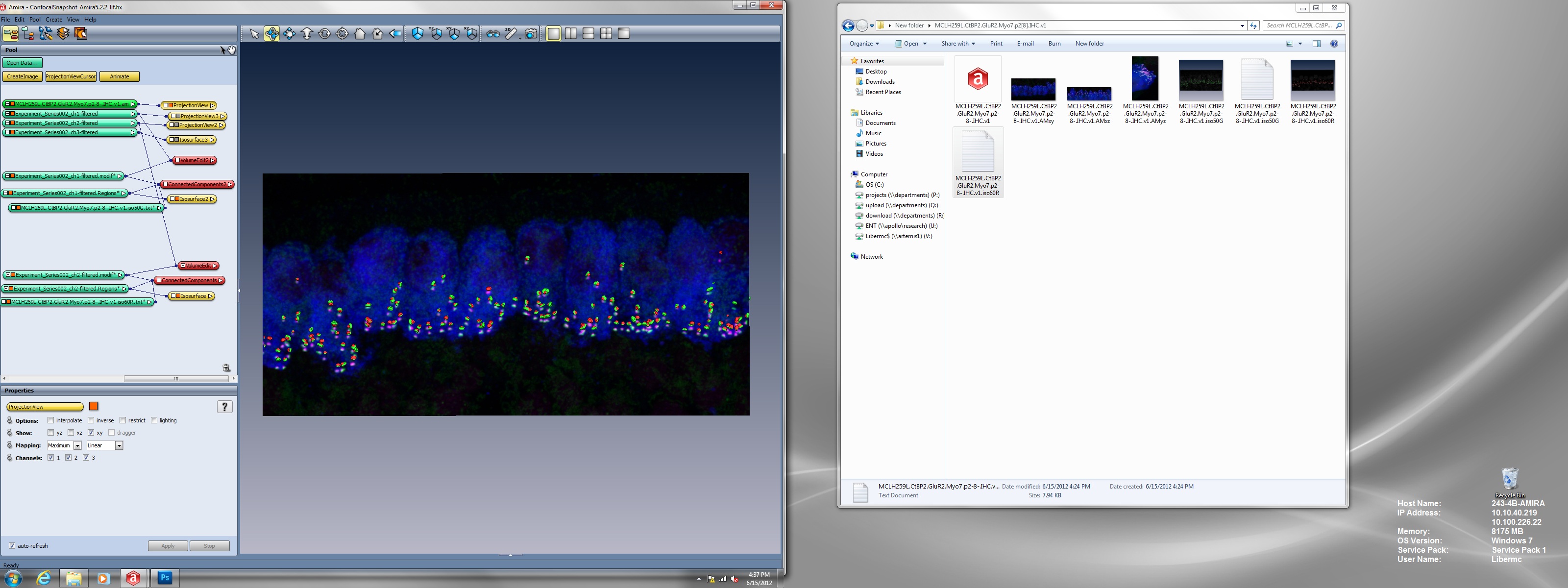

Supplement: Source code 1. — Amira and Blob Projection software. DOI: http://dx.doi.org/10.7554/eLife.03564.015 [file elife03564s001.zip › 5281_1_supp_83813_ndfs9t(1)/Amira screen shot.jpg]
